# Phase-Dependent Suppression of Beta Oscillations in Parkinson's Disease Patients

**DOI:** 10.1523/JNEUROSCI.1913-18.2018

**Published:** 2019-02-06

**Authors:** Abbey B. Holt, Eszter Kormann, Alessandro Gulberti, Monika Pötter-Nerger, Colin G. McNamara, Hayriye Cagnan, Magdalena K. Baaske, Simon Little, Johannes A. Köppen, Carsten Buhmann, Manfred Westphal, Christian Gerloff, Andreas K. Engel, Peter Brown, Wolfgang Hamel, Christian K.E. Moll, Andrew Sharott

**Affiliations:** ^1^MRC Brain Network Dynamics Unit, Department of Pharmacology, University of Oxford, Oxford OX1 3TH, United Kingdom,; ^2^Departments of Neurophysiology and Pathophysiology,; ^3^Neurology, and; ^4^Neurosurgery, University Medical Center Hamburg-Eppendorf, 20246 Hamburg, Germany,; ^5^Institute of Neurology, University College London, London WC1N 3BG, United Kingdom,; ^6^Sobell Department of Motor Neuroscience and Movement Disorders, UCL Institute of Neurology, London WC1E 6BT, United Kingdom, and; ^7^Nuffield Department of Clinical Neurosciences, John Radcliffe Hospital, University of Oxford, Oxford OX3 9DU, United Kingdom

**Keywords:** beta oscillations, clinical neurophysiology, deep brain stimulation, Parkinson's disease, subthalamic nucleus, synchrony

## Abstract

Synchronized oscillations within and between brain areas facilitate normal processing, but are often amplified in disease. A prominent example is the abnormally sustained beta-frequency (∼20 Hz) oscillations recorded from the cortex and subthalamic nucleus of Parkinson's disease patients. Computational modeling suggests that the amplitude of such oscillations could be modulated by applying stimulation at a specific phase. Such a strategy would allow selective targeting of the oscillation, with relatively little effect on other activity parameters. Here, activity was recorded from 10 awake, parkinsonian patients (6 male, 4 female human subjects) undergoing functional neurosurgery. We demonstrate that stimulation arriving on a particular patient-specific phase of the beta oscillation over consecutive cycles could suppress the amplitude of this pathophysiological activity by up to 40%, while amplification effects were relatively weak. Suppressive effects were accompanied by a reduction in the rhythmic output of subthalamic nucleus (STN) neurons and synchronization with the mesial cortex. While stimulation could alter the spiking pattern of STN neurons, there was no net effect on firing rate, suggesting that reduced beta synchrony was a result of alterations to the relative timing of spiking activity, rather than an overall change in excitability. Together, these results identify a novel intrinsic property of cortico-basal ganglia synchrony that suggests the phase of ongoing neural oscillations could be a viable and effective control signal for the treatment of Parkinson's disease. This work has potential implications for other brain diseases with exaggerated neuronal synchronization and for probing the function of rhythmic activity in the healthy brain.

**SIGNIFICANCE STATEMENT** In Parkinson's disease (PD), movement impairment is correlated with exaggerated beta frequency oscillations in the cerebral cortex and subthalamic nucleus (STN). Using a novel method of stimulation in PD patients undergoing neurosurgery, we demonstrate that STN beta oscillations can be suppressed when consecutive electrical pulses arrive at a specific phase of the oscillation. This effect is likely because of interrupting the timing of neuronal activity rather than excitability, as stimulation altered the firing pattern of STN spiking without changing overall rate. These findings show the potential of oscillation phase as an input for “closed-loop” stimulation, which could provide a valuable neuromodulation strategy for the treatment of brain disorders and for elucidating the role of neuronal oscillations in the healthy brain.

## Introduction

Neural oscillations play a fundamental role in normal brain processing by temporally coordinating activity within and across regions (Engel et al., 2001; Buzsáki and Draguhn, 2004). Dysfunctional communication resulting from an inability to properly modulate oscillatory activity, either through hypo- or hyper-synchrony, has been implicated in a number of neurological disorders (Schnitzler and Gross, 2005; Uhlhaas and Singer, 2006). Lesions, pharmacological treatments, and high-frequency stimulation can all be used to disrupt exaggerated rhythmic activity, however these manipulations often result in wide-spread effects on network activity. Being able to selectively control synchrony without disruption to other physiological activity has the potential to improve therapies and provide insight into its role in normal functioning.

Functional neurosurgery for Parkinson's disease (PD) offers a unique opportunity to study the generation, propagation and perturbation of neuronal oscillations in the human brain. The implantation of deep brain stimulation (DBS) electrodes allows for both recording and electrical stimulation of basal ganglia nuclei. Such experiments have clearly demonstrated that the loss of midbrain dopamine neurons leads to abnormally sustained and synchronized beta oscillations (15–30 Hz) across the cortex and basal ganglia (Cassidy et al., 2002; Levy et al., 2002; Kühn et al., 2005; Sharott et al., 2018). These oscillations are thought to be mechanistically involved in symptom manifestation by distorting communication between brain areas needed for initiation of voluntary movement (Brown, 2007; Engel and Fries, 2010; Dorval and Grill, 2014).

The amplitude of beta oscillations correlates with severity of akinetic/rigid symptoms (Kühn et al., 2006; Brown, 2007; Sharott et al., 2014; Neumann et al., 2016), and importantly their reduction following high-frequency (HF; ≥100 Hz) DBS positively correlates with motor improvement (Kühn et al., 2008; Ray et al., 2008; Zaidel et al., 2010). Although some have failed to demonstrate such a relationship (Blumenfeld et al., 2015), and a causal role in symptom generation is debated (Syed et al., 2012; Devergnas et al., 2014), recent studies suggest that beta activity is at least an effective biomarker for ongoing symptoms (Rosin et al., 2011; Little et al., 2013; Johnson et al., 2016).

Although effective, HF DBS is limited by stimulation-induced side effects (Hariz et al., 2008; Tripoliti et al., 2011; Castrioto et al., 2014) and partial efficacy (Little and Brown, 2012). Triggering bursts of HF stimulation only during periods of high-amplitude beta improves efficacy and reduces electrical energy delivered (Little et al., 2013); however, this could still disrupt physiological activity at timescales relevant for coding of movement (Amirnovin et al., 2004; Rodriguez-Oroz et al., 2005; Schrock et al., 2009; Lipski et al., 2017; Sharott et al., 2018).

A phase-dependent approach, where stimulation is timed to a certain phase of the ongoing beta oscillation, has the potential to more selectively dampen the oscillatory activity. The utility of such a strategy can be seen in controlling tremor, where stimulation is locked to a specific phase of the behavioral oscillation (Cagnan et al., 2017). In many neurological disorders, such as akinesia/rigidity in PD, where no peripheral oscillation provides a marker of symptom severity, it may be necessary to time stimulation based on neuronal oscillations (Rosin et al., 2011; Azodi-Avval and Gharabaghi, 2015; Holt et al., 2016; Meidahl et al., 2017; Moll and Engel, 2017). The approach is conceptually attractive, as it has the potential to modulate the timing of activity within and between structures, with less impact on gross excitability.

Using intraoperative electrophysiological recordings in PD patients, we demonstrate that there is a patient-specific phase of the subthalamic nucleus (STN) LFP beta oscillation at which consecutive pulses of electrical stimulation can suppress the amplitudes of local oscillations and network synchrony. These results provide the first evidence in humans for using the phase of a subcortical oscillation to more selectively control its amplitude, and opens up the possibility of using such an approach for neurological disorders with oscillatory pathologies and to test the mechanistic role of these activities in functional processes.

## Materials and Methods

This study was conducted in agreement with the Code of Ethics of the World Medical Association (Declaration of Helsinki, 1967) and was approved by the local ethics committee. All patients were previously diagnosed with advanced idiopathic PD and gave their informed consent to participate. Recordings were made intra-operatively from 10 patients undergoing awake surgery for bilateral implantation of DBS electrodes into the STN. Two patients were excluded from analysis for reasons discussed in the Results section.

### 

#### 

##### Patient information.

Recordings were made while simultaneously delivering stimulation in 10 patients (6 males, 4 females, average age: 62.1 years SD: 7.6 years). All patients had akinetic/rigid symptoms, had significant improvement of motor symptoms following levodopa intake as evaluated using the motor section (III) of the Unified Parkinson's Disease Rating Scale (UPDRS), displayed no major cognitive decline (evaluated using the Mattis Dementia Rating Scale; Mattis, 1988), and were awake during the surgical procedure. Participation in the study extended the surgical procedure by ∼15–30 min. Every effort was made to keep additional time to a minimum, and stress level was continuously monitored using a verbally administered numerical rating scale to ensure any prolongation had no effect on the patient's level of distress. Clinical details are summarized in Table 1.

**Table 1.** Patient details

Case	Age, years (gender)	Disease duration, years	Motor UPDRS OFF	Motor UPDRS ON	Preoperative anti-Parkinson drugs	Hoehn/Yahr score	Dominant side	Mattis dementia rating scale	Major symptoms	Included in analysis
1	72 (M)	6	33	13	Levodopa 650 g	3	Right	143	Akinetic, rigid	Yes
2	64 (M)	7	50	19	Levodopa 250 mg, Budipin, Piribedil, Rasagilin	3	Left	127	Akinetic, rigid	Yes
3	70 (F)	9	30	12	Levodopa 1350 mg	3/4	Left	137	Rigid, tremor	Yes
4	60 (M)	12	31	17	Levodopa 1000 mg	2	Right	141	Akinetic, rigid, tremor	Yes
5	68 (F)	8	18	13	Levodopa 500 mg	3	Right	139	Akinetic rigid	Yes
6	56 (F)	10	34	16	Levodopa 450 mg	2	Right	139	Akinetic rigid	Yes
7	57 (M)	17	33	16	Levodopa 1600 mg	3	Right	143	Akinetic, rigid, tremor	Yes
8	62 (F)	17	60	33	Levodopa 1400 mg, Apomorphine, Pramippexol	4	Left	137	Akinetic, rigid, tremor	Yes
9	53 (M)	22	63	9	Levodopa 600 mg, Benserazide 25 mg, Rasagiline 1 mg	3	Right	137	Akinetic, rigid, tremor	No
10	49 (M)	10	21	8	Levodopa 600 mg, Benserazide 25 mg, Ropinirole 32 mg, Safinamide 100 mg	2	Right	142	Akinetic, rigid	No

##### Surgical procedures.

Stereotaxic bilateral implantation of DBS electrodes into the STN was performed under local anesthesia. Surgical procedures and targeting details have been previously described (Hamel et al., 2003; Moll et al., 2014). Briefly, before surgery patients stopped taking all anti-parkinsonian medication overnight. Surgical planning of the electrode trajectories was based on fused images of CT and MRI scans acquired the day of surgery. The stereotaxic targeting of STN was approximated based on the following coordinates: 11–13 mm lateral, 1–3 mm inferior, and 1–3 mm posterior to the midcommissural point. The trajectory was altered to avoid major blood vessels, sulci, and ventricles. Low-dose procedural sedation and analgesia with remifentanil was stopped before the microelectrode mapping procedure.

##### Electrophysiological recording.

Microelectrode recordings were performed along three parallel tracks positioned in the central, anterior, and either lateral or medial positions of a BenGun arrangement (Neuro Omega, Alpha Omega). The central electrode was aimed at the anatomically planned target and was separated by 2 mm from outer electrodes anteriorly in the parasagittal plane and laterally in the coronal plane. STN borders could be readily delineated based on elevated background activity levels (Moran et al., 2006) and characteristic firing properties of STN neurons (Sharott et al., 2014). Both unit activity and local field potentials (LFPs) were recorded from the microelectrode contact. Unit activity was bandpass filtered between 0.6 and 6 kHz, amplified (×20,000), and sampled at 44 kHz, whereas LFPs were bandpass filtered between 0.00070 and 0.4 kHz and sampled at 1.375 kHz. Recordings were referenced to the uninsulated distal most part of the guide tube for the corresponding microelectrode (macrotip diameter ∼0.8 mm, length ∼1.5 mm, impedance <1 kΩ), located 3 mm above the microtip. EEG was recorded from scalp electrodes (needle electrodes) placed approximately at positions Fz, Cz, Pz (according to the international 10-20 system), referenced to the nose. Signals were amplified (×55,000), bandpass filtered between 0 and 0.3 kHz, and sampled at 1.375 kHz.

##### Electrical stimulation of the dorsal STN area.

**Figure 1.**
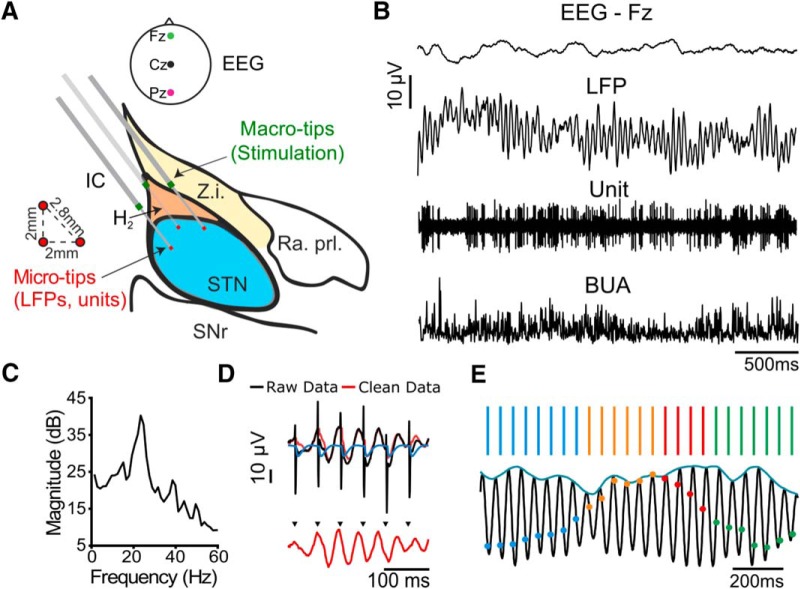
Corticosubthalamic recordings during beta frequency stimulation in parkinsonian patients. ***A***, Surgical setup, sagittal view of the subthalamic area, 11 mm lateral to the midline; (modified from Schaltenbrand and Bailey, 1959). Z.i., Zona incerta; IC, internal capsule; H_2_, field H_2_ of Forel; Ra. Prl., prelemniscal radiation; SNr, substantia nigra pars reticulata. Three microelectrodes were implanted using the central, anterior, and either lateral or medial trajectory in the BenGun arrangement. Stimulation was delivered through macro-tips located dorsal to the STN while LFPs and units were recorded from microtips within the STN. EEG was recorded from midline locations. ***B***, Example signals: EEG, LFP, unit activity, and BUA (generated using the unit channel). ***C***, Oscillations were detected from the spectral power of the LFP (example patient, 23 Hz peak, corrected for 1/f falloff). ***D***, A Kalman filter was used to generate an artifact free signal (red) using the raw signal (black) and a model of the average artifact (blue). ***E***, When the stimulus frequency was well matched to the peak beta frequency, consecutive cycles of stimulation at the same phase (blue, green, orange, or red) occurred coincidentally.

Bipolar, biphasic, stimulation pulses were delivered through the macroelectrode contacts of two electrodes while the microelectrode recording contact of the third electrode was within the STN. Stimulation parameters were as follows: total pulse width: 200 μs, 100 μs initial phase negative,100 μs positive phase; amplitude: 0.25–2 mA; constant current; stimulation time: 15–115 s, as permitted (>30 s in all but 1 patient). This resulted in stimulation being applied to the area immediately dorsal to the STN while LFPs and units were recorded from within the STN (Fig. 1*A*). Stimulation parameters were selected to modulate neuronal activity within the STN, while still allowing for a reliable LFP signal to be recovered from the recording electrode following stimulus artifact removal. Stimulation did not result in motor-evoked potentials.

Stimulation was applied at or near the peak beta frequency (beta-frequency stimulation) to determine effects of stimulation timing on beta oscillation amplitude. When well matched, stimulus pulses occurred at the same phase of the oscillation for at least two consecutive cycles (Fig. 1*E*). However, because of the natural variability in frequency and burst-like nature of the oscillation (Feingold et al., 2015; Tinkhauser et al., 2017), pulses drifted through different phases of the oscillation over the entire recording (Brittain et al., 2013; Cagnan et al., 2013). Only patients whose peak beta oscillation frequency was within 5 Hz of the stimulation frequency were included in analysis to ensure consecutive cycles of stimulation occurring at the same phase.

##### Spike train processing.

Spike trains (single and multiunit) were separated from background activity using standard spike sorting procedures *post hoc* (Spike2, Cambridge Electronic Design; Mallet et al., 2008a,b), including template matching, principal component analysis, and supervised clustering. When a cluster was not separable, spike trains were defined as multiunits. Firing rates during stimulation were compared with rates before the onset of the first stimulus pulse at a given stimulus amplitude and depth. For the calculation of rates during stimulation, a 2.5 ms window following each pulse during which spikes could not be detected because of the resulting artifact was removed. The Wilcoxon's signed rank test was used to evaluate effects of beta-frequency stimulation on firing rate.

##### Stimulus artifact removal.

Data were analyzed offline using MATLAB (MathWorks). A linear interpolation was used to remove sharp electrical artifacts in signals. To remove stimulus-evoked artifacts seen in the LFP a Kalman filter approach was used (Morbidi et al., 2007; Fig. 1*D*). The Kalman filter is a recursive approach which predicts the current state of the system and uses noisy measurements as feedback to update the prediction at each sample point. Briefly, we assume the recorded LFP is a summation of the unstimulated signal and the stimulus artifact. An autoregressive model was fit to a segment of unstimulated data and a transfer function model was fit to the average stimulus-evoked artifact. The Kalman filter was then implemented and results used to estimate the artifact-free signal without phase distortion.

##### Spectral power analysis.

To evaluate overall effects of beta frequency stimulation on LFP beta power, spectra were normalized to the total power between 5 and 45 Hz and expressed as percentage of total power (%). Power between 0 and 5 Hz and >45 Hz was eliminated to avoid contamination by movement and mains noise. The Wilcoxon's signed rank test was used to evaluate statistical effects of stimulation on beta power.

##### Instantaneous phase and amplitude estimation.

To estimate the phase and amplitude of the beta oscillation, signals were bandpass filtered ±3 Hz around the peak beta frequency using a second-order Butterworth filter with zero-phase digital filtering to preserve the true phase of the signal. The Hilbert transform was then used to estimate the instantaneous phase and envelope of the oscillation. Phase is defined as ϕ(*t*) = $\text{atan}\left(\frac{v\left(t\right)}{H\left(v\left(t\right)\right)}\right)$, where *v*(*t*) is the filtered LFP signal and H(*v*(*t*)) is the Hilbert transform of *v*(*t*). The amplitude envelope is defined as *A*(*t*) = $\sqrt{(v{\left(t\right)}^{2}+H{\left(v\left(t\right)\right)}^{2}}$.

##### Instantaneous effects of stimulus phase.

To assess how stimulus pulses occurring at a certain phase of the beta oscillation affect the beta amplitude envelope, stimulus phase was divided into eight overlapping phase bins ¼ of a cycle wide. The percentage change in median envelope over the cycle following the stimulus pulse was compared with the median envelope of the entire signal. Surrogate results were generated by sampling an unstimulated portion of data at the stimulation frequency. The Kruskal–Wallis test was used to assess phase-dependent effects on beta amplitude in both the surrogate and stimulation conditions. Stimulation effects for each phase bin were compared with surrogates using the Wilcoxon rank sum test (FDR-corrected for multiple comparisons). Boxplots throughout are plotted with the central dot representing the median and box edges as the 25th and 75th percentiles. Outliers are plotted individually and defined as outside *q*_75_ − *w* * (*q*_75_ − *q*_25_) and *q*_25_ + w * (*q*_75_ − *q*_25_), where *q*_25_ and *q*_75_ are the 25th and 75th percentiles, respectively, and *w* is the maximum whisker length (∼±2.7σ).

##### Cumulative effects of stimulus phase.

To evaluate cumulative phase-dependent effects of stimulation on beta amplitude, periods where stimulation occurred at the same phase (8 overlapping bins, ¼ of a cycle wide) coincidentally were used. As phase was not being tracked in real-time, the chances of observing further stimuli occurring within the same phase bin decreased as the number of stimuli increased (i.e., 5 consecutive stimuli in the same phase bin occurred less often than 3). If there were fewer than five occurrences of one, two, three, four, five, or six consecutive pulses delivered at a specific phase throughout the entire recording, this occurrence was eliminated from analysis. For each patient, suppressing and amplifying bins were defined as the phase bins leading to the maximum suppression and amplification of the oscillation envelope in the LFP respectively. The Kruskal–Wallis test and *post hoc* Dunn's pairwise tests (10 comparisons) were used to assess the significance of consecutive pulses at the amplifying and suppressing phase bin for each patient as well as across the group (Hochberg and Benjamini, 1990). Subsequently, to determine how precision of the defined stimulus phase affected beta amplitude modulation, the width of the amplifying and suppressing phase bins were widened and narrowed around the mean.

To investigate whether discrete phase changes accompany beta amplitude modulation, we looked at the percentage of stimulus pulses that led to phase slips when stimulating at either the suppressing or amplifying phase for consecutive cycles. Phase slips were identified when the instantaneous frequency (derivative of the unwrapped phase) exceeded two SDs above the mean, indicating a discontinuity in the oscillation phase (Pikovsky et al., 2001). In an effort to avoid naturally occurring phase slips, only those occurring within 15 ms following the stimulus pulse were counted.

To evaluate the effects of consecutive stimuli occurring at the defined amplifying and suppressing phase on local neuronal activity, background unit activity (BUA) was used. The BUA represents neuronal activity of a population of neurons around the recording contact, distinct from single and multiunit activity (Moran and Bar-Gad, 2010). To generate the BUA signal, large amplitude spikes (3 SD above the mean) were removed from the unit microelectrode recording by replacing a window from 1 ms before to 3 ms after each spike with a random 4 ms spike-free segment of the same recording. The signal was then low-pass filtered at 300 Hz (third-order Butterworth, zero-phase digital filtering), rectified, and downsampled to 1.375 kHz (Moran et al., 2008; Moran and Bar-Gad, 2010; Sharott et al., 2017). The BUA was bandpass filtered and analyzed for cumulative phase-dependent effects of stimulation on signal amplitude as described for the LFP.

Midline EEG signals were used to assess corticosubthalamic synchrony during stimulation at the defined amplifying and suppressing phase. EEG signals were bandpass filtered between 0.001 and 0.1 kHz to remove the contribution of slow drifts and high-frequency activity, and notch filtered between 0.049 and 0.051 kHz, to remove line noise. To evaluate the phase relationship between the STN (LFP) and cortex (EEG), instantaneous phase of the EEG signal was determined as described for the LFP (second-order Butterworth bandpass filter, Hilbert transform). The phase synchrony index (PSI) between the two signals was then calculated over epochs of three consecutive stimuli occurring at the suppressing or amplifying phase (Stam et al., 2007). The PSI is defined as follows:

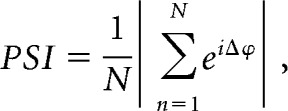
 where *N* is the length of the segment (3 cycles), and Δϕ is the phase difference between the cortical and LFP signal (calculated using the Circular Statistics toolbox; Berens, 2009). PSI values range from 0 to 1, with 1 representing a constant phase difference between the two signals.

##### Surrogates.

Surrogates were used to ensure that neither the natural variability of the signal nor analysis techniques could produce similar effects to stimulation. To generate surrogates, identical analysis was run on a time-matched unstimulated portion of the recording sampled at the stimulation frequency. New suppressing and amplifying phases were identified for each surrogate to guard against the possibility that (1) there is some regularity in the underlying beta amplitude that consistently corresponds to the occurrence of stimulus epochs for a specific phase, and (2) that presorting and subsequently grouping bins across patients leads to minor but significant effects. Stimulation effects were compared with surrogate effects using the Wilcoxon ranked sum test with the false discovery rate (FDR) correction for multiple comparisons (5 comparisons; Benjamini and Hochberg, 1995).

In addition to the surrogates described, further analysis was performed to verify phase-dependent beta modulation could not be explained by suppressing or amplifying stimuli consistently starting at vulnerable portions of the oscillation by chance. The amplitude at the time of the first pulse for suppressing and amplifying epochs was analyzed to assess whether there were any significant differences. Furthermore, the phase stability of the STN LFP oscillation was assessed for epochs of three consecutive amplifying or suppressing stimuli by calculating entropy values from histograms of cycle lengths during the epochs of interest (as determined by zero crossings of the filtered signal). Cycle lengths were divided into *B* equally spaced bins 1 ms wide. An entropy bias term was used to correct for the different number of suppressing and amplifying epochs (Roulston, 1999): *Bias* = $\frac{B-1}{2N}$, where N is the total number of cycles. Entropy was normalized by the maximum possible entropy: *Entropy* = $\frac{\sum_{i=1}^{B}P\left(i\right)\ln P\left(i\right)-Bias}{B\ln\frac{1}{B}}$, where *P*(*i*) is the probability that a given cycle length occurred in bin *i*. A perfectly stable signal with a fixed frequency would have an entropy value of zero.

##### Experimental design and statistical analysis.

Statistical analysis was performed using MATLAB. Phase-dependent effects were evaluated using the Kruskal–Wallis test (with Dunn post-tests for multiple comparisons, 10 comparisons). When comparing cumulative effects to surrogates, the Wilcoxon ranked sum test was used, correcting for multiple comparisons using the FDR (Benjamini and Hochberg, 1995). Significance between two groups (i.e., firing rate under stimulation ON vs stimulation OFF conditions) was assessed using the Wilcoxon ranked sum test (reported with test statistic, *W*). Nonparametric tests were chosen because of the small sample sizes.

## Results

The overarching aim of this study was to determine whether stimulation occurring at a specific phase relative to the ongoing beta oscillation could produce short-latency effects on the amplitude of pathophysiological activity in the STN. Electrical stimulation near the peak beta frequency was applied dorsal to the STN while STN LFPs and unit activity, together with EEG, were recorded in 10 PD patients (Fig. 1*A–D*). When stimulation and oscillation frequency were well matched, pulses could occur at a consistent phase of the LFP beta oscillation for two or more cycles coincidentally, while drifting through all phases over the entire recording (Fig. 1*E*). Two patients were excluded from analysis; one did not have a significant beta oscillation (spectral power evaluation), and in the other the stimulus and oscillation frequencies were >5 Hz apart, preventing the stimulus phase from staying consistent over consecutive cycles. In the eight patients included in analysis, the average oscillation frequency was 19 ± 5 Hz, and the stimulation frequency was 2.75 ± 1.75 Hz different from the peak beta frequency.

### Establishing stimulation parameters to investigate phase-dependent effects

**Figure 2.**
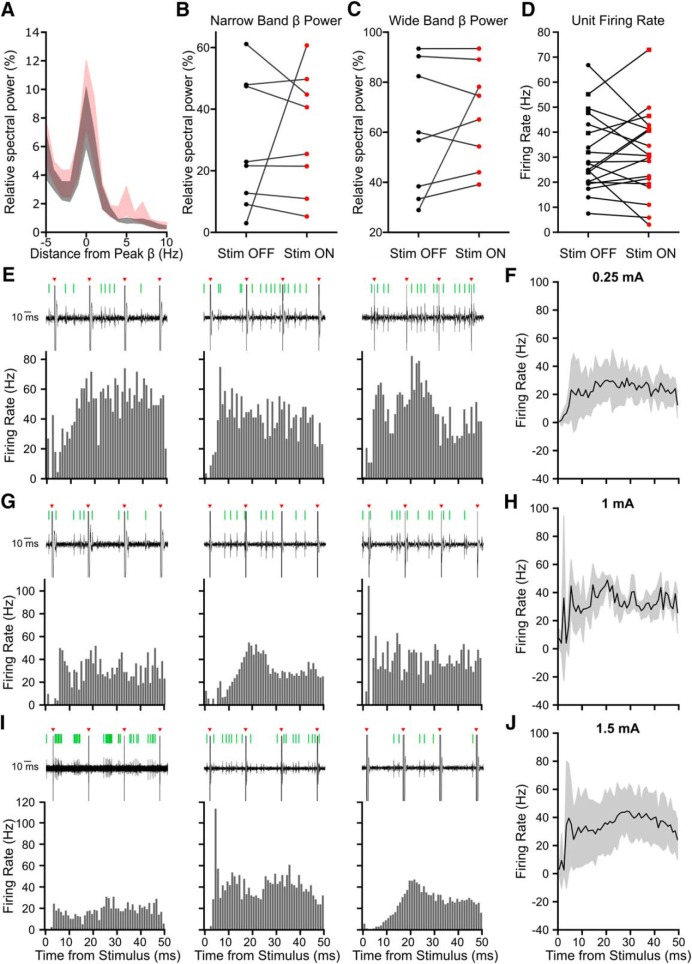
Beta frequency stimulation does not consistently modulate STN activity on the order of seconds but does modulate STN unit firing patterns. Beta frequency stimulation did not affect beta power or firing rate over the entire recording but did alter spike timing, as seen in peristimulus time histograms (PSTHs). ***A***, Average ± SEM power spectra aligned to the peak beta frequency shows spectral activity calculated across the entire recording did not change significantly with beta frequency Stim ON across eight patients. A prominent beta peak was seen in Stim OFF (black) and Stim ON (red) conditions. ***B***, There was no significant difference in peak beta power (peak beta frequency ± 3 Hz) relative to 5–45 Hz with stimulation (red; *n* = 8 patients, *W* = 65, *p* = 0.798, Wilcoxon ranked sum test). ***C***, There was no significant difference in total wide band beta frequency power (8–35 Hz) relative to 5–45 Hz with stimulation (*n* = 8 patients, *W* = 62 *p* = 0.574, Wilcoxon ranked sum test). ***D***, There was no significant difference in firing rates of putative subthalamic units between Stim OFF and Stim ON periods (*n* = 19 units, *W* = 498, *p* = 0.953, Wilcoxon ranked sum test). Circles indicate cells classified as single units, squares multiunits. ***E***, ***G***, ***I***, PSTHs, using 1 ms wide bins, from nine example STN units (single or multiunits) across seven patients. Beta frequency stimulation was applied at 0.25 mA (***E***), 1 mA (***G***), and 1.5 mA (***I***). Spikes were detected from microelectrode recordings in the STN; representative examples of raw unit data during three consecutive electrical stimuli are shown above each PSTH (black, raw trace; red arrow, stimulation; green line, detected spike). ***F***, ***H***, ***J***, Average (±SD) PSTH in response to 0.25 mA (7 units, 3 patients), 1 mA (3 units, 2 patients), and 1.5 mA (8 units, 4 patients) stimulation.

Beta frequency stimulation has been shown to worsen akinetic/rigid motor symptoms (Fogelson et al., 2005; Chen et al., 2011) and inhibit STN firing in PD patients (Milosevic et al., 2018). However, to prevent confounding the interpretation of changes in oscillatory activity, we required a stimulation protocol that could modulate STN activity without leading to gross changes over timescales of seconds. Following stimulus artifact removal, the spectral content of the LFP was similar to that observed without stimulation (Fig. 2*A*). Stimulation did not increase either peak (±3 Hz; Fig. 2*B*) or wide band (8–35 Hz; Fig. 2*C*) beta power relative to total power between 5 and 45 Hz in seven of eight patients (*n* = 8 patients; peak: *W* = 65, *p* = 0.798; wide band: *W* = 62 *p* = 0.574; Wilcoxon ranked sum test). Stimulation increased beta power in one patient, thus it is possible to increase beta power with 20 Hz stimulation in this setup. However, as this only occurred in one patient, it was unlikely to affect phase-dependent results of the group. Additionally, stimulation did not consistently alter the firing rate of STN units with respect to the unstimulated baseline (*n* = 19 units, W = 498, *p* = 0.953, Wilcoxon ranked sum test; Fig. 2*D*). Nevertheless, stimulation could lead to short-latency excitation or inhibition of spiking, often followed by further multiphasic responses (Fig. 2*E*, *G*, *I*). Because unit responses were variable, when averaged there was no visible effect (Fig. 2*F*,*H*,J). These results demonstrate that the stimulation protocol used could alter the spike timing of individual STN neurons, without gross changes in firing rate or LFP beta power.

### Phase-dependent modulation of beta amplitude

**Figure 3.**
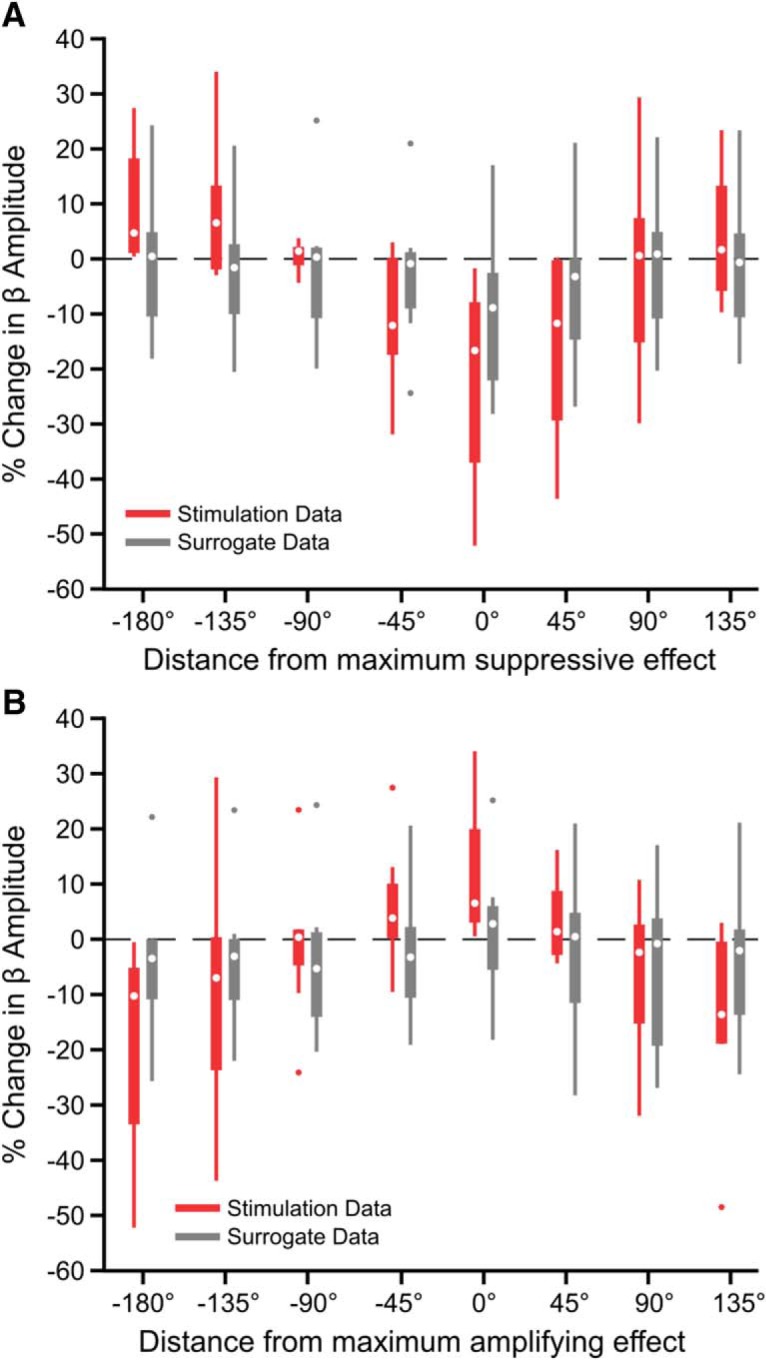
Phase-dependent effects of single stimuli on beta amplitude did not exceed variability of the unstimulated LFP. Without taking into consideration the phase of past stimuli, phase-dependent effects of stimulation on beta amplitude (red) did not exceed effects seen using a time-matched unstimulated portion of the data sampled at the stimulation frequency (gray) across eight patients. All stimulus pulses were grouped into eight overlapping phase bins, ¼ of a cycle wide. Phase-dependent effects of stimulation on beta amplitude were seen when phase bins were aligned (***A***) to the bin showing the maximum beta suppression for each patient (χ^2^ = 28.74, *p* = 0.0002, Kruskal–Wallis test) as well as (***B***) to the bin showing the maximum amplifying effect for each patient. Surrogates did not show a significant phase-dependent trend for either (***A***; χ^2^ = 4.9, *p* = 0.673, Kruskal–Wallis test or (***B***; χ^2^ = 3.39, *p* = 0.847, Kruskal–Wallis test). However, stimulus-induced modulation of the beta amplitude was not significantly different from modulation seen using surrogates for any phase bin in either alignment (*p* > 0.05, Wilcoxon ranked sum test). Data are shown using a boxplot where the central dot is the median and box edges are the 25th and 75th percentiles. Outliers are plotted individually and defined as outside *q*_75_ − *w* * (*q*_75_ − *q*_25_) and *q*_25_ + *w* * (*q*_75_ − *q*_25_) where *q*_25_ and *q*_75_ are the 25th and 75th percentiles, respectively, and w is the maximum whisker length.

Using the established stimulus parameters, we first investigated transient modulation of STN LFP beta oscillation amplitude by grouping all stimuli into eight overlapping phase bins, without accounting for the stimulus phases on previous cycles. While there was a significant phase-dependent trend in amplitude modulation both when bins were grouped relative to the maximum suppressing (χ^2^ = 28.74, *p* = 0.0002, Kruskal–Wallis test; Fig. 3*A*) and maximum amplifying bins (χ^2^ = 25.61, *p* = 0.0006, Kruskal–Wallis test; Fig. 3*B*), effects were not significantly different from those seen by sampling an unstimulated portion of the recording at the stimulation frequency (*p* > 0.05, *n* = 8 patients, Wilcoxon ranked sum test, FDR corrected, 8 comparisons). This suggests there were no phase-dependent effects of single stimuli on LFP beta amplitude beyond what was seen in the natural variability of the signal.

**Figure 4.**
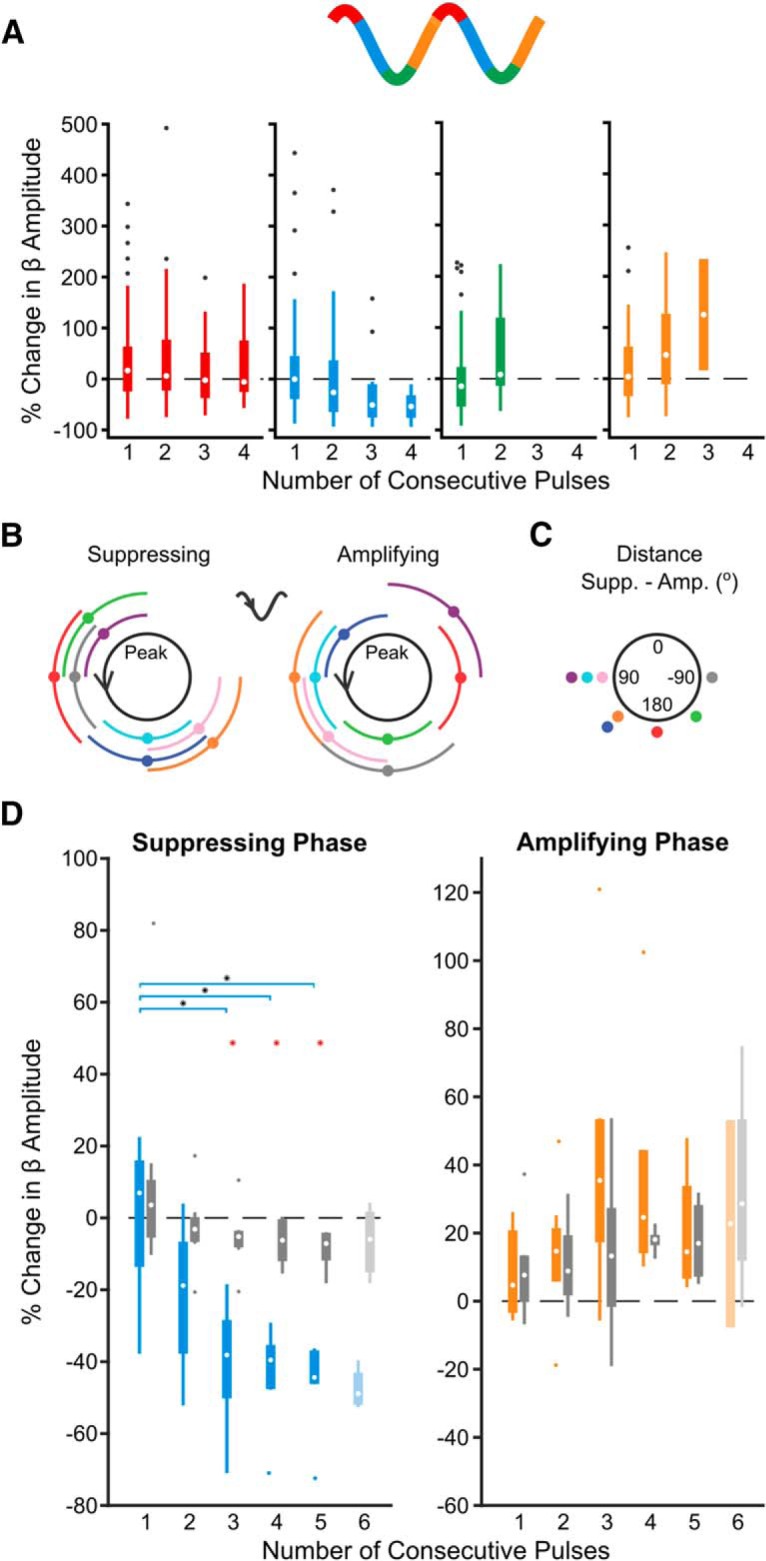
Consecutive phase-consistent stimulus pulses modulate beta oscillation amplitude. Stimulation at the patient-specific suppressing phase for at least three consecutive cycles led to suppression of STN LFP beta oscillations across eight patients. ***A***, Percentage change in beta oscillation amplitude from the median after consecutive cycles of stimuli occurring at a consistent phase is shown for four phase bins in an example subject. In this subject, amplitude suppression was seen after three consecutive cycles of stimuli delivered on the descending phase of the oscillation (blue), whereas amplification was seen after three consecutive cycles of stimuli delivered on the ascending phase (orange). Stimuli delivered at alternative phases (red, green) did not result in modulation of the beta amplitude. ***B***, Suppressing and amplifying phase bins for each patient. ***C***, Phase difference between the amplifying and suppressing phase bin for each patient. ***D***, Median suppressing (blue) and amplifying (orange) effects were grouped across eight patients. The percentage change in beta amplitude was compared with surrogate effects (identical analysis on unstimulated segment of the recording sampled at the stimulus frequency; gray). Beta suppression was dependent on the number of consecutive stimuli delivered at the suppressing phase of the oscillation (χ^2^ = 17.38, *p* = 0.00160, Kruskal–Wallis test), whereas beta amplification was not (χ^2^ = 6.12, *p* = 0.190, Kruskal–Wallis test). As six consecutive stimuli were only observed in four of the eight patients at the suppressing phase and two of eight patients at the amplifying phase (indicated by lighter boxes), these was not included in the Kruskal–Wallis test. Horizontal lines with black asterisks indicate differences between groups (*post hoc* Dunn test to correct for multiple comparisons, *p* ≤ 0.05). Red asterisks indicate stimulation effects significantly different from surrogates (*p* ≤ 0.05, Wilcoxon ranked sum test, FDR-corrected).

Next, we investigated cumulative phase-dependent effects on beta oscillations by using epochs during which the stimulation phase was consistent within a quarter of a cycle for two or more consecutive cycles. In individual subjects, consecutive pulses at a given phase could either suppress (suppressing phase) or amplify (amplifying phase) the beta amplitude over the following cycle compared with the median (Fig. 4*A*). Importantly, similar numbers of consecutive stimuli delivered at alternative phases did not result in a change in amplitude. The phase bins leading to suppression and amplification were specific to each individual patient (Fig. 4*B*), but their difference was always at least 90° (Fig. 4*C*).

In the case of consecutive stimulation at the suppressing phase, reduction in beta amplitude was dependent on the number of consecutive stimuli across patients (χ^2^ = 17.38, *p* = 0.00160, Kruskal–Wallis test; Fig. 4*D*). The mean percentage reduction went from 21.8% after two consecutive stimuli to 46.8% after five. Suppression was significantly beyond what was seen using surrogates following the third (*p* = 0.0031), fourth (*p* = 0.0063), and fifth (*p* = 0.0072) consecutive pulses (first pulse: *p* = 0.959; second pulse: *p* = 0.1037; Wilcoxon rank sum test, FDR-corrected, 5 comparisons). In individual patients, stimulation at the suppressing phase resulted in a significant suppressive trend in 5 of 8 patients (*p* < 0.05, *p* = 6.88e−9, *p* = 2.33e-10, *p* = 0.277, *p* = 0.366, *p* = 1.52e−18, *p* = 0.126, *p* = 0.00260, *p* = 4.37e−13, Kruskal–Wallis test), whereas no individual surrogate showed such a trend (*p* > 0.05, *p* = 0.996, *p* = 0.906, *p* = 0.641, *p* = 0.511, *p* = 0.972, *p* = 0.443, *p* = 0.128, *p* = 0.998, Kruskal–Wallis test). The strength of suppression was inversely correlated with the relative spectral power at beta frequencies calculated across the entire recording (*r*^2^ = 0.504, *F* = 6.09, *p* = 0.049, linear regression analysis), suggesting it may be more difficult to modulate stronger synchrony.

In contrast to suppressive effects, amplification of the beta oscillation was not dependent on the number of consecutive stimuli (χ^2^ = 6.12, *p* = 0.190, Kruskal–Wallis test; Fig. 4*D*) and was not significantly greater than surrogates (first pulse: *p* = 0.886; second pulse: *p* = 0. 886; third pulse: *p* = 0. 886; fourth pulse: *p* = 0. 886; fifth pulse: *p* = 0. 886, Wilcoxon ranked-sum test, FDR-corrected, 5 comparisons). At the individual level, a significant amplification trend was only seen in 2 of 8 patients (*p* < 0.05, *p* = 1.217e−5, *p* = 1.874e−8, *p* = 0.842, *p* = 0.0591, *p* = 0.118, *p* = 0.362, *p* = 0.150, *p* = 0.268, Kruskal–Wallis test), whereas no individual surrogate showed such a trend (*p* < 0.05, *p* = 0.594, *p* = 0.935, *p* = 0.719, *p* = 0.170, *p* = 0.762, *p* = 0.766, *p* = 0.162, *p* = 0.153, Kruskal–Wallis test). The degree of beta amplification was inversely correlated with the relative spectral beta power over the entire recording (*r*^2^ = 0.540, *F* = 7.05, *p* = 0.038, linear regression analysis), suggesting it may be more difficult to further amplify the already exaggerated beta signal.

**Figure 5.**
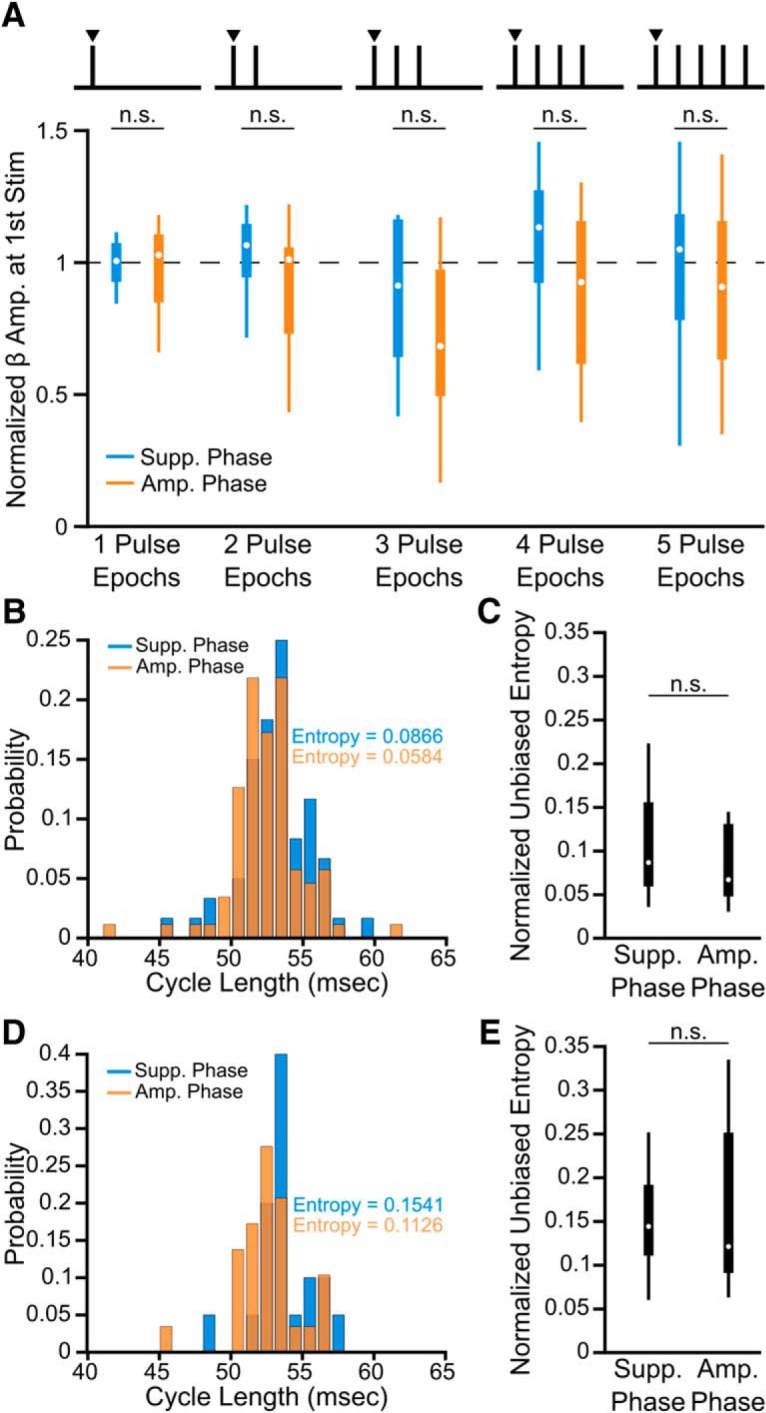
Beta-amplitude effects cannot be explained by differences in initial amplitude or phase stability. ***A***, Beta amplitude (normalized to the median) at the time of the first pulse during epochs of consecutive stimuli did not differ between amplifying or suppressing phases (1 Pulse Epochs: *p* = 0.879; 2 Pulse Epochs: *p* = 0.742; 3 Pulse Epochs: *p* = 0.742; 4 Pulse Epochs: *p* = 0.742; 5 Pulse Epochs: *p* = 0.828; Wilcoxon rank sum test, FDR-corrected, 5 comparisons). ***B***, Normalized histogram of cycle lengths over entire epochs of three consecutive pulses at the suppressing or amplifying phase for an example subject. ***C***, Across eight patients there was no significant difference in the phase stability of the beta signal over epochs of three consecutive suppressing or amplifying stimuli as determined from entropy measures derived from cycle length histograms (*W* = 73, *p* = 0.645, Wilcoxon rank sum test). ***D***, Normalized histogram of cycle lengths for the initial cycle of epochs of three consecutive suppressing or amplifying stimuli for an example patient. ***E***, Across eight patients, there was no significant difference in the phase stability of the initial cycle of three consecutive suppressing or amplifying pulse epochs (*W* = 69, *p* = 0.959, Wilcoxon rank sum test).

Additional analysis was performed to ensure epochs of suppressing stimuli were not consistently occurring at a vulnerable period of the beta oscillation by chance. Although unlikely to be phase-dependent, one concern would be if suppressing epochs consistently started at a peak beta amplitude, where a decrease occurs naturally over subsequent cycles. A second concern would be if suppressing epochs consistently occurred during prolonged periods of unstable, low-amplitude beta. However, epochs of consecutive suppressing stimuli did not start at a significantly different amplitude than epochs of amplifying pulses (1 Pulse Epochs: *p* = 0.879; 2 Pulse Epochs: *p* = 0.742; 3 Pulse Epochs: *p* = 0.742; 4 Pulse Epochs: *p* = 0.742; 5 Pulse Epochs: *p* = 0.828; Wilcoxon rank sum test, FDR corrected, 5 comparisons), and importantly did not show any trend when moving from 1 to 5 consecutive stimulus pulses (Suppressing: χ^2^ = 1.98, *p* = 0.739; Amplifying: χ^2^ = 2.67, *p* = 0.61; Kruskal–Wallis test; Fig. 5*A*). Furthermore, for epochs of three consecutive stimuli at the amplifying or suppressing phase, there was no significant difference in the phase stability over the three cycles (*W* = 73, *p* = 0.645, Wilcoxon rank sum test; Fig. 5*B*,*C*) or for the initial cycle (*W* = 69, *p* = 0.959, Wilcoxon rank sum test; Fig. 5*D*,*E*).

**Figure 6.**
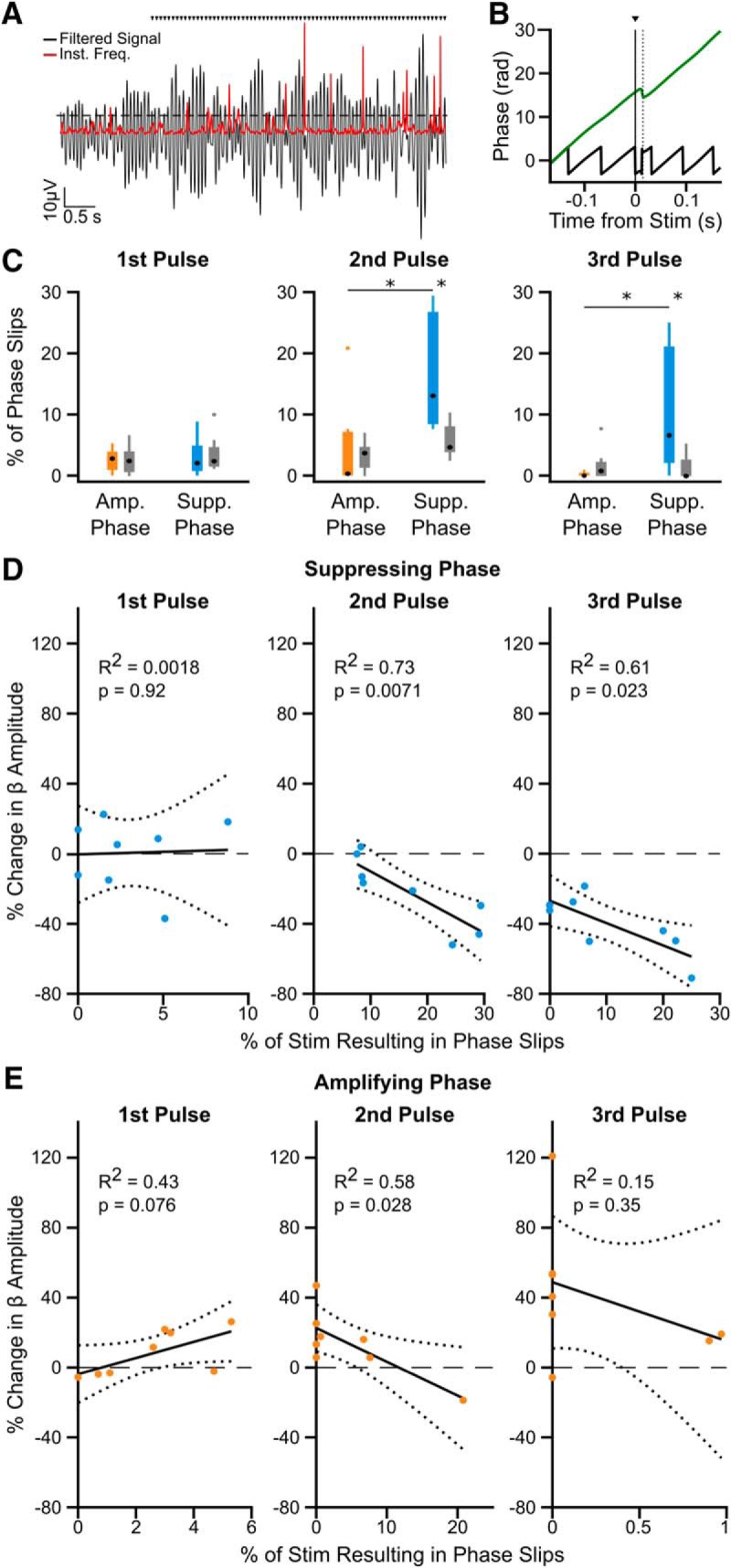
Increased phase slips in the beta oscillation following consecutive pulses at the suppressing phase correlates with amplitude reduction. ***A***, Phase slips were defined when the instantaneous frequency (red) of the beta filtered signal (black) crossed 2 SD above the mean (dotted line), indicating a phase discontinuity in the oscillatory signal. Black triangles indicate stimulation pulses. ***B***, Example phase slip within 15 ms following a stimulus pulse (dotted line), seen in both the unwrapped phase (green) and phase (black) of the oscillation. ***C***, The percentage of stimulus pulses with phase slips occurring within 15 ms following the first (left), second (middle), and third (right) consecutive pulse at the amplifying (orange) or suppressing (blue) phase. Significantly more phase slips are seen after two (*W* = 94, *p* = 0.00420) and three (*W* = 90, *p* = 0.0120) pulses at the suppressing phase than at the amplifying phase (Wilcoxon ranked sum test), and when compared with surrogates generated by running identical analysis on a time-matched unstimulated segment of the recording (2 pulses: *W* = 92, *p* = 0.0096; 3 pulses: *W* = 88, *p* = 0.0297; Wilcoxon ranked sum test). (Asterisks indicate *p* <= 0.05) ***D***, The percentage of phase slips occurring after the second (middle) and third (right) pulse at the suppressing phase correlates with the reduction in beta amplitude (*F* = 15.984, *p* = 0.00710; *F* = 9.252, *p* = 0.0230, linear regression), but not following the first pulse (left; *F* = 0.0110, *p* = 0.92, linear regression). Note maximum *x*-axis values are variable. ***E***, The percentage of phase slips only correlates with beta oscillation amplification after the second consecutive pulse at the amplifying phase (middle; *F* = 8.234, *p* = 0.0280, linear regression), not after the first (left) or third (right). Note the change in *x*-axis values compared with those in ***D***, as less phase slips occur at the amplifying phase.

It has been suggested that phase-dependent modulation of neuronal oscillations may rely on stimulation-induced changes to the phase of the oscillation (Wilson and Moehlis, 2014; Azodi-Avval and Gharabaghi, 2015; Holt et al., 2016). To address whether this mechanism could apply here, we investigated whether more phase slips, indicating discontinuities in the oscillation phase, were seen in the 15 ms following consecutive cycles of stimulation at the suppressing or amplifying phase (reported as a percentage of stimulus pulses; Fig. 6*A*,*B*). In line with amplitude effects, there was no difference in the percentage of phase slips following the first stimulus at the suppressive compared with amplifying phase (*n* = 8 patients; *W* = 68, *p* = 1.00; Wilcoxon ranked sum test; Fig. 6*C*). However, after the second and third consecutive pulse, significantly more phase slips are seen following stimuli arriving at the suppressing phase compared with the amplifying phase (*n* = 8 patients; second pulse: *W* = 94, *p* = 0.00420; third pulse: *W* = 90, *p* = 0.0120; Wilcoxon ranked sum test; Fig. 6*C*). In fact, almost no phase slips are seen following the third pulse at the amplifying phase, suggesting the oscillation is robust. The number of phase slips after consecutive cycles of suppressing stimulation was significantly different from surrogates (*n* = 8 patients; second pulse: *W* = 92, *p* = 0.0096; third pulse: *W* = 88, *p* = 0.0297; Wilcoxon ranked sum test). Furthermore, after the second and third stimulus at the suppressing phase, the percentage of phase slips correlates with the reduction in beta amplitude (*n* = 8 patients; second pulse: *R*^2^ = 0.730, *F* = 15.984, *p* = 0.00710; third pulse: *R*^2^ = 0.610, *F* = 9.252, *p* = 0.0230; linear correlation), but not after the first pulse (*n* = 8 patients, *R*^2^ = 0.0018, *F* = 0.0110, *p* = 0.920; linear correlation; Fig. 6*D*). At the amplifying phase, there was a significant inverse correlation with beta amplification, but only following the second stimulus pulse (*n* = 8 patients; first pulse: *R*^2^ = 0.433, *F* = 4.573, *p* = 0.0763; second pulse: *R*^2^ = 0.580, *F* = 8.234, *p* = 0.0280; third pulse: *R*^2^ = 0.147, *F* = 1.032, *p* = 0.349; linear correlation; Fig. 6*E*). These results are consistent with stimulation at the suppressing phase advancing or delaying the neuronal oscillation.

### Increased phase-specificity enhances cumulative phase-dependent suppression of beta amplitude

**Figure 7.**
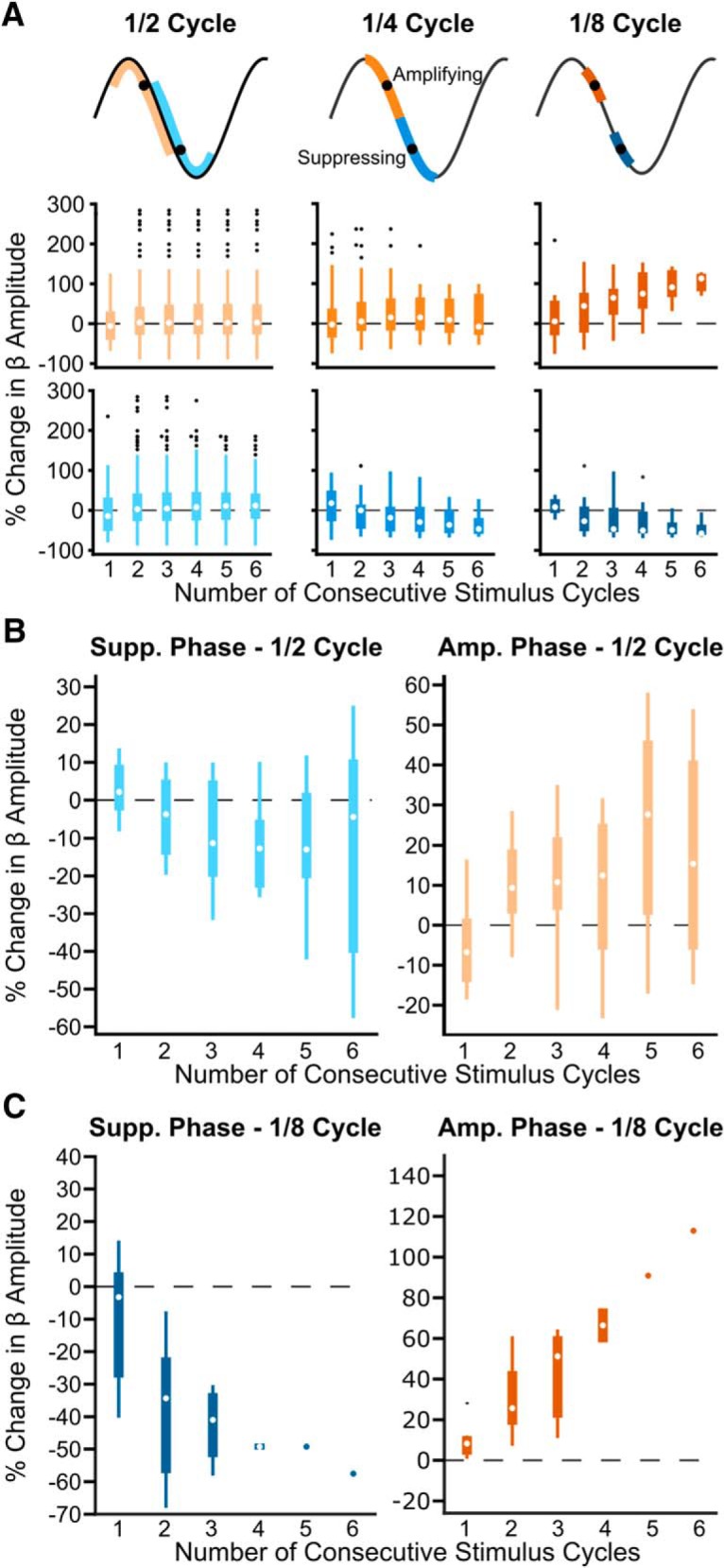
Phase precision of the stimulus pulse affects the strength of beta amplitude modulation. Increased stimulus phase precision leads to stronger modulation of beta amplitude. ***A***, Example patient showing both enhanced suppression and amplification of beta amplitude when using narrower phase bins. Bins were widened or narrowed around the mean suppressing and amplifying phase (black dots). Stimulus phase was defined as follows: (left) half the oscillation cycle, (middle) ¼ the oscillation cycle, (right) ⅛ the oscillation cycle. Blue hues represent the suppressing phase; orange hues represent the amplifying phase. Amplitude modulation was only dependent on number of consecutive pulses when using ⅛ the oscillation cycle (amplifying phase: 2 bins, χ^2^ = 1.24, *p* = 0.941; 4 bins, χ^2^ = 2.05, *p* = 0.842; 8 bins, χ^2^ = 9.89, *p* = 0.0423; suppressing phase, 2 bins, χ^2^ = 5.23, *p* = 0.388; 4 bins, χ^2^ = 10.99, *p* = 0.0517; 8 bins, χ^2^ = 12.62, *p* = 0.0272, Kruskal–Wallis test). ***B***, Median suppressing and amplifying effects using phase bins half a cycle wide across eight patients. Neither beta suppression (χ^2^ = 6.33, *p* = 0.276) nor amplification (χ^2^ = 6.62, *p* = 0.250) was dependent on the number of consecutive stimuli (Kruskal–Wallis test). This is in contrast to results seen in Figure 5 where narrower phase bins (¼ cycle wide) were used. ***C***, Median suppressing and amplifying effects using phase bins ⅛ cycle wide across eight patients. Because it was unlikely to see three or more consecutive stimulus cycles using the narrower phase bins, <5 patients were included in many of these bins. For the suppressing phase: first pulse, *n* = 8 patients; second pulse, *n* = 8 patients; third pulse, *n* = 4 patients; fourth pulse, *n* = 2 patients; fifth pulse, *n* = 1 patient; sixth pulse, *n* = 1 patient. For the amplifying phase: first pulse, *n* = 8 patients; second pulse, *n* = 6 patients; third pulse, *n* = 3 patients; fourth pulse, *n* = 2 patients; fifth pulse, *n* = 1 patient; sixth pulse, *n* = 1 patient.

To understand how precisely the stimulus pulse must hit the suppressing or amplifying phase to modulate beta amplitude, we performed the same analysis using wider or narrower phase bins. In Subject 3 both enhanced suppression (χ^2^ = 12.62, *p* = 0.0272, Kruskal–Wallis test) and amplification (χ^2^ = 9.89, *p* = 0.0423, Kruskal–Wallis test) were seen when stimulus pulses occurred in a narrower window (⅛ cycle) around the average suppressing and amplifying phase; however, neither suppression (χ^2^ = 5.23, *p* = 0.388, Kruskal–Wallis test) nor amplification (χ^2^ = 1.24, *p* = 0.941, Kruskal–Wallis test) occurred using a wider window (½ cycle; Fig. 7*A*). Similar effects can be seen when looking across the group (Fig. 7*B*,*C*). Neither significant suppression (χ^2^ = 6.33, *p* = 0.276, Kruskal–Wallis test) nor amplification (χ^2^ = 6.62, *p* = 0.250, Kruskal–Wallis test) was seen when the stimulus phase was consistent within ½ a beta cycle across patients (Fig. 7*B*). Stimulation frequency within 1 Hz of the peak beta frequency made it possible to see up to 6 consecutive stimulus cycles at a given phase when using narrower bins (⅛ cycle; Fig. 7*A*). However, as the frequencies were not as well matched across all patients, fewer than five subjects showed at least four consecutive cycles at the suppressing and amplifying bins. Therefore, rigorous group statistics were not possible using narrower phase bins, but data can we seen in Figure 7*C*. Overall, these results suggest at least a quarter cycle stimulus phase precision is needed to see beta amplitude modulation, but increased precision may lead to even larger effects, particularly for amplification.

### Phase-dependent suppression of beta amplitude was dependent on stimulation amplitude

**Figure 8.**
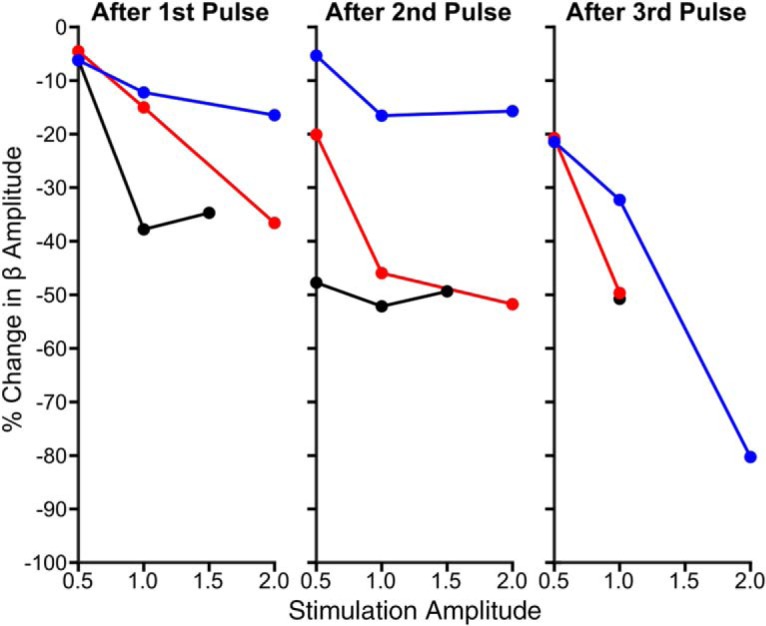
Phase-dependent suppression of beta oscillations is dependent on stimulation amplitude. Percentage change in beta oscillation amplitude after the first (left), second (middle), and third (right) consecutive stimulus pulse at the suppressing phase as a function of stimulus amplitude is plotted for three patients. After each stimulus, the decrease in beta amplitude was stronger when using 1 versus 0.5 mA. Further suppression could be seen when the stimulus amplitude was increased to 2 mA; however, effects were not as consistent.

To test whether phase-dependent suppression of beta oscillations was dependent on stimulation amplitude, three amplitudes (0.5, 1, and 1.5 or 2 mA) were applied while maintaining a consistent recording/stimulation location in three patients. Across patients, stronger suppression was seen using 1 mA stimulation than 0.5 mA after one, two, or three consecutive pulses at the suppressing phase (Fig. 8). As the stimulus amplitude was increased further suppressive effects became more variable, with maximum suppression achieved seemingly reaching a plateau in 2 of the 3 patients.

### Phase-dependent suppression of rhythmic STN output

**Figure 9.**
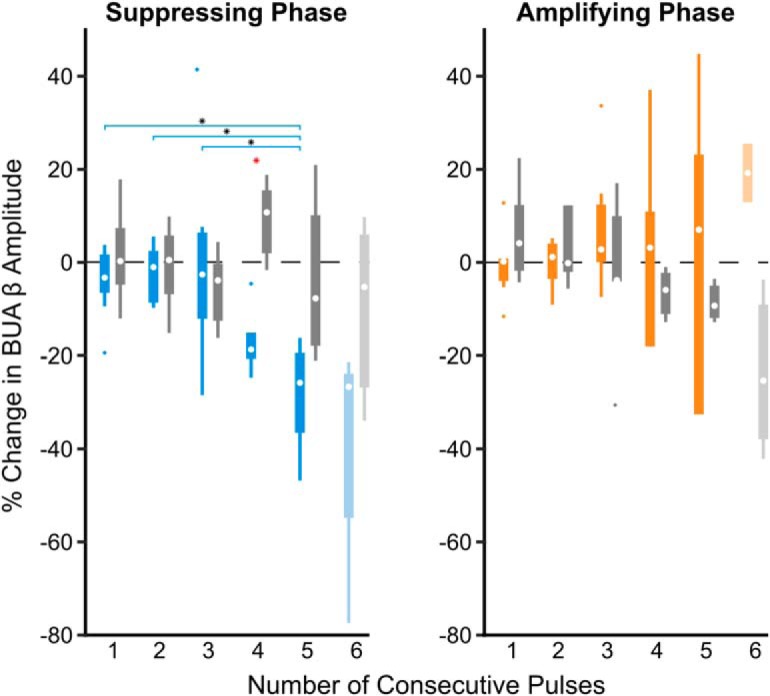
Consecutive stimulus pulses at the suppressing phase leads to suppression of the beta synchronous STN unit activity. BUA was filtered around the peak beta frequency detected in the LFP. Median percentage changes in the oscillation amplitude (compared with the median) following stimulation at the suppressing (blue) and amplifying (orange) phase were grouped across eight patients. Suppression of beta-frequency activity in the BUA was dependent on the number of consecutive stimuli delivered at the suppressing phase of the LFP oscillation (χ^2^ = 15.06, *p* = 0.00460, Kruskal–Wallis test), whereas amplification was not (χ^2^ = 1.65, *p* = 0.799, Kruskal–Wallis test). As six consecutive stimuli were only observed in 4 of the 8 patients at the suppressing phase and two of eight patients at the amplifying phase (indicated by lighter boxes), these was not included in the Kruskal–Wallis test. Horizontal lines with black asterisks indicate differences between groups (corrected for multiple comparisons using *post hoc* Dunn test, *p* ≤ 0.05). Red asterisks indicate stimulation effects significantly different from surrogates (*p* ≤ 0.05, Wilcoxon ranked sum test, FDR-corrected).

We next investigated whether stimulating at the amplifying or suppressing phase, as defined by the STN LFP, led to concurrent modulation of spiking output. As locations with strong LFP oscillations were prioritized, stable single units were not recorded for the duration of stimulation in all positions. Thus, BUA was used as a measure of output from the local population of neurons, as it could be analyzed at every location included in the LFP analysis. When consecutive stimuli occurred at the suppressing phase of the LFP, the amplitude of beta frequency rhythmic activity in the BUA simultaneously decreased across patients (χ^2^ = 15.06, *p* = 0.00460, Kruskal–Wallis test; Fig. 9). Instead of seeing suppressive effects by the second cycle, a decrease in BUA rhythmic activity was not seen until the fourth consecutive cycle, where there was a 18.7% reduction compared with the median, which was significantly different from surrogate results (*p* = 0.0397, Wilcoxon rank sum test, FDR-corrected, 5 comparisons). Consecutive stimulus pulses at the amplifying phase of the LFP did not result in significant enhancement of rhythmic activity in the BUA (χ^2^ = 1.65, *p* = 0.799, Kruskal–Wallis test; *p* > 0.05 when comparing to surrogates, Wilcoxon rank sum test, FDR-corrected, 5 comparisons; Fig. 9). These results indicate that stimulating at the suppressing phase of the LFP results in a concurrent decrease in synchronous beta-frequency output of STN neurons, and could therefore modulate activity in downstream structures and the wider network.

### Phase-dependent suppression led to decreased corticosubthalamic beta synchronization

**Figure 10.**
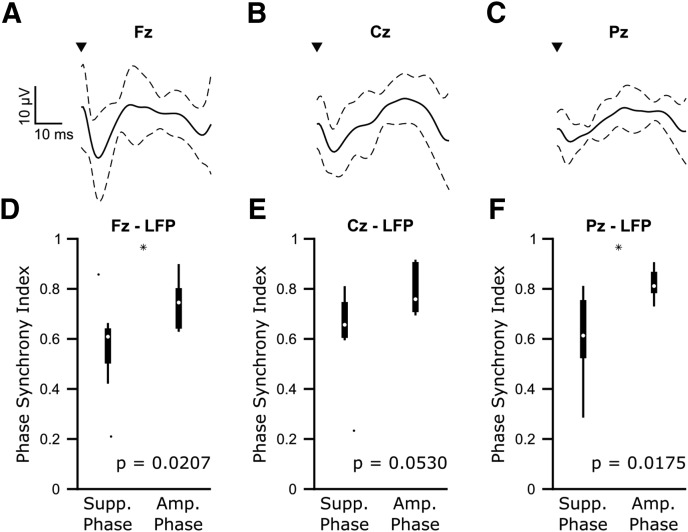
Phase-dependent suppression of subthalamic beta oscillations is associated with lower corticosubthalamic beta synchronization than phase-dependent amplification. ***A***–***C***, Average (±SD) cortical-evoked responses from frontal/midline EEG (Fz, Cz, and Pz) locations) across eight patients for Fz and seven patients for Cz and Pz. EEG signals were bandpass filtered 1–100 Hz to remove any slow drift, and notch filtered 49–51 Hz to remove line noise. ***D***–***F***, Median phase synchrony index between the STN LFP and frontal/midline EEG channels over three cycles of consecutive stimuli occurring at the suppressing and amplifying phases across eight patients (Fz) or seven patients (Cz, Pz). Stimulating at the suppressing phase resulted in significantly lower corticosubthalamic phase synchrony for Fz-LFP (*n* = 8, *W* = 46, *p* = 0.0207, Wilcoxon ranked sum test) and Pz-LFP (*n* = 7, *W* = 34, *p* = 0.0175, Wilcoxon ranked sum test, *p* < 0.05 indicated by black asterisks). Cz-LFP (*n* = 7, *W* = 37, *p* = 0.0530).

Previous studies have demonstrated that corticosubthalamic beta synchronization is correlated with the severity of akinetic/rigid symptoms (Sharott et al., 2018). Using EEG recordings, we examined whether consecutive cycles of stimulation at the amplifying and suppressing phase determined from the STN LFP led to differences in corticosubthalamic synchronization. In line with previous work (Ashby et al., 2001; Eusebio et al., 2009; Walker et al., 2012; Kumaravelu et al., 2018), stimulation pulses led to evoked potentials in midline EEG channels (Fig. 10*A–C*). Although this demonstrates cortical activity could be modulated using our stimulation protocol, stimulation at the amplifying and suppressing LFP phases did not result in simultaneous changes in cortical beta amplitude (Fz: *n* = 8 patients, amplifying: χ^2^ = 7.18, *p* = 0.13, suppressing: χ^2^ = 0.69, *p* = 0.95; Pz: *n* = 7 patients, amplifying: χ^2^ = 1.79, *p* = 0.77, suppressing: χ^2^ = 1.03, *p* = 0.90; Cz: *n* = 7 patients, amplifying: χ^2^ = 2.6, *p* = 0.63, suppressing: χ^2^ = 0.06, *p* = 1.00; Kruskal–Wallis test). However, the phase alignment between midline EEGs and the STN LFP was less consistent during three cycles of stimulation at the suppressing phase than at the amplifying phase (Fz-LFP: *n* = 8 patients, *W* = 46, *p* = 0.0207; Cz-LFP: *n* = 7 patients, *W* = 37, *p* = 0.0530; Pz-LFP: *n* = 7 patients, *W* = 34, *p* = 0.0175; Wilcoxon rank sum test; Fig. 10*D–F*). Importantly, the differences between amplifying and suppressing effects of corticosubthalamic synchrony were not seen using surrogates (Fz-LFP: *n* = 8 patients, *W* = 65, *p* = 0.955; Cz-LFP: *n* = 7 patients, *W* = 57, *p* = 0.295; Pz-LFP: *n* = 7 patients, *W* = 53, *p* = 0.628; Wilcoxon rank sum test), suggesting the result is not trivial. These findings demonstrate that phase-dependent stimulation has network-level effects on beta synchrony.

## Discussion

In this study we show that electrical stimulation delivered to a specific phase of the subthalamic beta oscillation in patients with PD results in suppression of local and network level pathophysiological activity. Remarkably, stimulus pulses arriving at the suppressing phase over multiple consecutive cycles reduced the beta amplitude by >40% across patients. This provides the first evidence that beta synchrony can be modulated depending on the input phase, a property that could be useful in developing more targeted stimulation strategies to reduce pathological neural oscillation while sparing disparate physiological activity in PD and other brain disorders.

### Potential mechanisms of stimulation

Any electrical stimulation of the brain, including high-frequency DBS, affects multiple neuronal elements in the vicinity of the stimulating electrode (McIntyre et al., 2004). Although the novel stimulation setup in this study allowed us to recover and analyze the underlying beta oscillation, the electrical current spread was likely different from conventional DBS. Because of the dorsal stimulating position, together with the horizontal orientation of the electric field (as opposed to the vertical orientation with conventional DBS electrodes), current was potentially delivered to multiple neuronal populations and fiber tracts (internal capsule, zona incerta, and fields of Forel) containing excitatory corticosubthalamic and inhibitory pallido-subthalamic axons (Hamani et al., 2004). Stimulation of these elements could lead to both orthodromic effects in the STN (and other targets) and antidromic effects at the source of the afferent fibers. Current may also have spread to the STN itself, but given the bipolar configuration it was likely more concentrated in these dorsal areas. Thus, as with therapeutic DBS, modulation of STN activity in our configuration likely occurred through a variety of direct and indirect mechanisms that cannot be fully delineated.

### Effects of 20 Hz stimulation on STN activity

The potential combination of stimulation effects could explain the variance in the multiphasic responses evoked in STN spiking activity, including both short latency (<10 ms) excitation and inhibition. Such multiphasic responses result from an integration of excitatory and inhibitory afferents with the pacemaker currents that drive the spontaneous firing of STN neurons (Nambu et al., 2002; Magill et al., 2004; Wilson and Bevan, 2011). While neuronal activity in the STN was clearly influenced by the stimulation, it did not produce a consistent modulation in a particular direction of magnitude or frequency. This contrasts with studies suggesting 20 Hz stimulation worsens bradykinesia (Brown et al., 2004; Chen et al., 2007), and should therefore amplify beta oscillations. Here, we did not see gross changes to beta power in response to 20 Hz stimulation, but it is important to note that results may be different when using higher amplitude pulses or a stimulation setup more similar to conventional DBS.

### Phase-dependent amplitude suppression relies on consecutive pulses

In PD, single STN neurons lock to a specific phase of the ongoing beta oscillation in the LFP (Kühn et al., 2005), which reflects synchronized membrane currents of a local population of neurons. In contrast, the BUA must predominantly reflect spiking activity due to exclusion of low (>300 Hz) frequencies (Moran et al., 2008). LFP signals are therefore more indicative of synaptic input to the neurons, whereas the BUA signal reflects the output (Sharott et al., 2017). Based on these assumptions, our results show that stimulation at consecutive pulses of the suppressing phase can reduce the oscillatory input to and output from the STN.

Stimulus pulses delivered to neural oscillators can result in advances or delays in the oscillator depending on the stimulus phase (Ermentrout and Chow, 2002; Smeal et al., 2010; Farries and Wilson, 2012). In line with this theory, stimulation at the suppressing phase of the beta oscillation resulted in more disruptions to the phase following the second and third pulse. While this could be due to the oscillation becoming less stable with amplitude reduction, epochs of three consecutive suppressing and amplifying stimuli showed similar phase stability. Within the STN, a phase shift in beta oscillatory input may alter or reflect the reliability of the recruitment of STN neurons to the cortical oscillation. Studies in experimental animals have demonstrated that the synchronization observed in the STN extends across the entire corticobasal ganglia network (Goldberg et al., 2002; Deffains et al., 2010; Sharott et al., 2017). Advancing or delaying STN oscillations could thus disrupt the temporal relationship between downstream brain regions, decoupling the network as a whole.

The necessity for consecutive pulses to achieve amplitude suppression could be because our stimulus amplitudes were too low to see instantaneous effects. Indeed, stronger suppression with fewer pulses was seen using higher stimulation amplitudes. Alternatively, disrupting the temporal relationships between oscillators within and between nodes of the network may require several cycles to decouple the circuit. Regardless, one key aspect in developing improved DBS protocols is reducing the current to prevent spread outside the target. Our results suggest delivering low-amplitude pulses at the suppressing phase is sufficient to disrupt network activity. Phase-dependent amplification was relatively weak, although could be more powerful using narrower phase bins. This may have been because the already heightened state of beta oscillations is more difficult to further enhance than suppress. Given that amplifying ongoing beta oscillations would potentially worsen symptoms, this property could be therapeutically useful.

The variability of suppressing and amplifying phases relative to the STN beta oscillation across patients could be explained by the heterogeneity in electrical stimulation effects, differences in recording location, or patient-specific differences. Although mean phase of the LFP at which STN units fire is relatively constant (Weinberger et al., 2006; Mallet et al., 2008b; Sharott et al., 2018), variance of stimulation mechanisms across patients might change the stimulation phase needed to disrupt synchronization between STN neurons. Alternatively, as beta oscillations arise in a complex network, response to a given perturbation may depend on other parameters, such as coupling strength and plastic changes of the network, which would likely vary between patients. Current results suggest any clinical application would require the suppressing phase to be calculated empirically, which could be achieved through the methods used here, or using some simple measure to predict the optimal phase, such as those proposed in various studies (Wilson and Moehlis, 2014; Azodi-Avval and Gharabaghi, 2015; Holt et al., 2016).

### Implications for therapy

Advancements to DBS algorithms have been aimed at improving efficacy and reducing the amount of current delivered to limit side effects and conserve battery power (Adamchic et al., 2014; Wilson and Moehlis, 2014; Brocker et al., 2017). Closed-loop approaches using unit activity (Rosin et al., 2011) or beta amplitude (Little et al., 2013) to control stimulation have been effective in achieving some of these aims. Oscillation phase may offer a more appealing feedback signal for a number of reasons. First, unlike units, LFP oscillations can be chronically recorded, even during unimpeded movement (Rosa et al., 2015). Second, delivering a stimulus pulse timed to the oscillation phase may better preserve physiological activity at timescales relevant for coding of movement. Finally, other oscillation-based measures implicated in motor symptoms, such as phase amplitude coupling of high-frequency activities to beta (de Hemptinne et al., 2013), would likely be disrupted by suppressing the carrier frequency.

### Limitations

Although the correlation between beta power and movement impairment is well established (Kühn et al., 2006; Brown, 2007; Sharott et al., 2014; Neumann et al., 2016), causality has not been definitively demonstrated. Therefore, it will be important to show that phase-dependent suppression has behavioral effects. The phase drifting approach only allowed for 50–300 ms epochs of phase-locked stimulation. Thus, stimulation driven active phase tracking during a precise motor task would likely be necessary to collect a sufficient amount of behavioral data at amplifying and suppressing phases. This would require a dedicated device capable of minimizing delays between phase detection and stimulus delivery, and online stimulus artifact removal to ensure that the ongoing oscillation, rather than the stimulation artifact, is being tracked. Using such a device in this setting would require justification for requisite ethical and safety approval, which is greatly enhanced by the results presented here.

### Conclusions

Our findings suggest that tracking the phase of ongoing beta oscillations and delivering stimulus pulses only on the suppressing phase could be an effective closed-loop strategy in PD. The increased number of consecutive stimulation cycles possible with active phase tracking could further enhance suppressive effects. While active phase-locked stimulation has been applied to low-frequency neural oscillations in the hippocampus (Siegle and Wilson, 2014) and low-frequency peripheral oscillations (Brittain et al., 2013; Cagnan et al., 2017), to our knowledge, this approach has not yet been successfully implemented at frequencies >10 Hz in humans. Implementing active phase tracking of parkinsonian beta oscillations presents challenges not only because of the higher frequency signal, but also because oscillations tend to occur in bursts (Tinkhauser et al., 2017) and are on the order of 1 μV. Overall, however, the results presented here provide strong evidence in support of exploring such an approach in the future for brain disorders where abnormal oscillations are a pathophysiological feature.
